# Comparison of noninvasive pulse transit time estimates as markers of blood pressure using invasive pulse transit time measurements as a reference

**DOI:** 10.14814/phy2.12768

**Published:** 2016-05-27

**Authors:** Mingwu Gao, N. Bari Olivier, Ramakrishna Mukkamala

**Affiliations:** ^1^ Department of Electrical and Computer EngineeringMichigan State UniversityEast LansingMichigan; ^2^ Department of Small Animal Clinical SciencesMichigan State UniversityEast LansingMichigan

**Keywords:** Cuff‐less blood pressure, PPG, pulse arrival time, pulse transit time, pulse wave velocity

## Abstract

Pulse transit time (PTT) measured as the time delay between invasive proximal and distal blood pressure (BP) or flow waveforms (invasive PTT [I‐PTT]) tightly correlates with BP. PTT estimated as the time delay between noninvasive proximal and distal arterial waveforms could therefore permit cuff‐less BP monitoring. A popular noninvasive PTT estimate for this application is the time delay between ECG and photoplethysmography (PPG) waveforms (pulse arrival time [PAT]). Another estimate is the time delay between proximal and distal PPG waveforms (PPG‐PTT). PAT and PPG‐PTT were assessed as markers of BP over a wide physiologic range using I‐PTT as a reference. Waveforms for determining I‐PTT, PAT, and PPG‐PTT through central arteries were measured from swine during baseline conditions and infusions of various hemodynamic drugs. Diastolic, mean, and systolic BP varied widely in each subject (group average (mean ± SE) standard deviation between 25 ± 2 and 36 ± 2 mmHg). I‐PTT correlated well with all BP levels (group average *R* ^2^ values between 0.86 ± 0.03 and 0.91 ± 0.03). PPG‐PTT also correlated well with all BP levels (group average *R* ^2^ values between 0.81 ± 0.03 and 0.85 ± 0.02), and its *R* ^2^ values were not significantly different from those of I‐PTT. PAT correlated best with systolic BP (group average *R* ^2^ value of 0.70 ± 0.04), but its *R* ^2^ values for all BP levels were significantly lower than those of I‐PTT (*P* < 0.005) and PPG‐PTT (*P* < 0.02). The pre‐ejection period component of PAT was responsible for its inferior correlation with BP. In sum, PPG‐PTT was not different from I‐PTT and superior to the popular PAT as a marker of BP.

## Introduction

Pulse transit time (PTT) is the time it takes for the pressure or flow wave to propagate between two arterial sites. PTT measured as the time delay between invasive proximal and distal blood pressure or flow (BP or BF) waveforms (henceforth referred to as invasive PTT [I‐PTT]) has been shown to correlate well with acute changes in BP over a wide physiological BP range (Geddes et al. 1981; Pruett et al. 1988; Ochiai et al. 1999; Gao et al. 2014). PTT estimated as the time delay between noninvasive proximal and distal arterial waveforms could therefore permit convenient tracking of BP changes. Indeed, noninvasive PTT estimates are being widely pursued at present for cuff‐less BP monitoring (Mukkamala et al. 2015).

The most popular noninvasive PTT estimate has been the time delay between ECG and photoplethysmography (PPG) waveforms (henceforth referred to as pulse arrival time [PAT]) (Mukkamala et al. 2015). PAT has shown reasonably good correlation with systolic BP (Mukkamala et al. 2015). However, the major concern is that PAT not only includes PTT but also the pre‐ejection period (PEP), which varies with cardiac electrical and mechanical properties. Another obvious, but hardly investigated, estimate is the time delay between proximal and distal PPG waveforms (henceforth referred to as PPG‐PTT) (Young et al. 1995; Chen et al. 2009). While this estimate eliminates the PEP concern, the timing of the PPG waveform, which indicates pulsatile blood volume, may not coincide with BP and BF waveforms due to viscoelastic delays, wave reflection, and/or other factors.

Despite the intense interest, the capabilities and limitations of these and other noninvasive PTT estimates in tracking BP changes are not yet fully understood. As outlined in (Mukkamala et al. 2015), few studies have assessed noninvasive PTT estimates as markers of BP over a wide physiological BP range. Furthermore, few studies have compared different noninvasive PTT estimates in terms of their ability to correlate with BP. Lastly, few studies have assessed noninvasive PTT estimates as markers of BP while using I‐PTT as a reference, which may establish the upper bound on performance.

In this study, an animal model was used to compare PAT, PPG‐PTT, and I‐PTT in terms of tracking acute BP changes over a wide physiological BP range. The results showed that PPG‐PTT was not significantly different from I‐PTT and superior to the popular PAT as a marker of BP.

## Materials and Methods

### Data collection

Experiments were performed in six healthy swine (30–40 kg). Each animal was studied on two separate days using procedures approved by the Michigan State University All‐University Committee on Animal Use and Care.

Chronic instrumentation was installed during a sterile procedure as follows. Anesthesia was induced with an intravenous injection of propofol (2.2–6.6 mg/kg) and maintained with inhaled isoflurane (1.5–2.5%), and mechanical ventilation was instituted. A left lateral thoracotomy was performed. An ultrasonic flow probe was placed around the ascending aorta for measurement of a proximal BF waveform (A‐series, Transonic Systems, NY). The chest was evacuated and closed in layers, with the cable tunneled subcutaneously and exteriorized between the scapulae. The animal was then allowed several days for recovery.

Following the recovery period, anesthesia was likewise induced and maintained, and mechanical ventilation was instituted and adjusted to keep end‐tidal CO_2_ at 40–45 mmHg. Skin electrodes were positioned for measurement of the ECG waveform (ECG100C, Biopac, CA); a micromanometer‐tipped catheter was inserted into a femoral artery for measurement of a distal BP waveform (SPC‐320, Millar Instruments, TX); an infrared, transmission‐mode PPG clip was attached to the tongue (in the first four subjects) or an infrared, reflectance‐mode PPG clip was attached to the skin on the neck (in the last two subjects) for measurement of a proximal PPG waveform (PPG100C, Biopac); and another infrared, reflectance‐mode PPG clip was attached to the skin a few centimeters distal to the opposite femoral artery for measurement of a distal PPG waveform. Additional instruments were also placed to address aims unrelated to this study. The analog transducer outputs were interfaced to a personal computer via an analog‐to‐digital conversion system (MP150, Biopac). The measurements were then recorded at a sampling rate of 500 Hz over the course of 150–250 min during baseline conditions and intravenous infusions of dobutamine, diltiazem, phenylephrine, nitroprusside, and norepinephrine. Various infusion rates were employed followed by recovery periods.

### Data analysis

**Figure 1:**
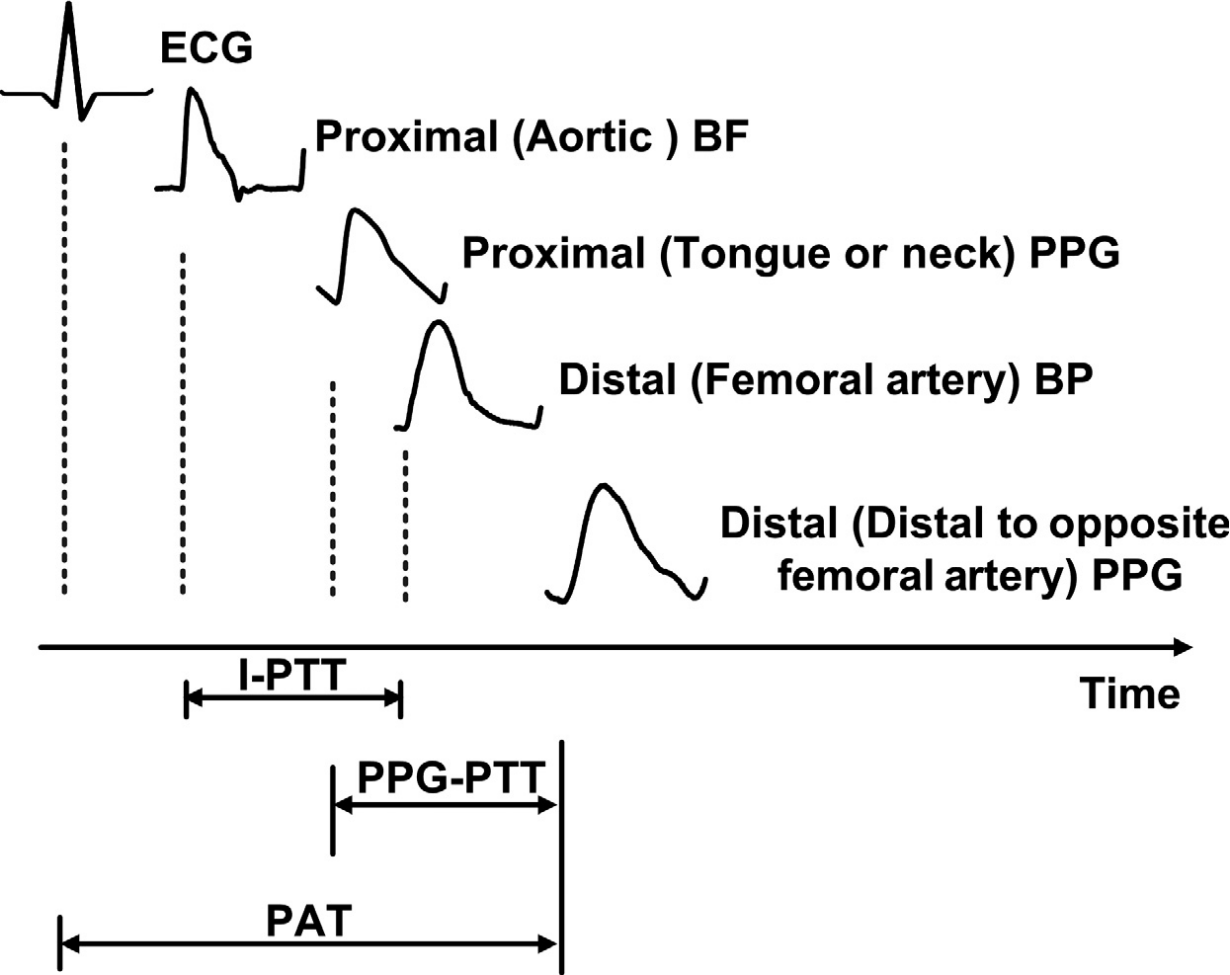
Determination of invasive pulse transit time (I‐PTT), photoplethysmography‐pulse transit time (PPG‐PTT), and pulse arrival time (PAT) from arterial and ECG waveforms measured from six swine. The proximal waveform for PPG‐PTT was obtained using a tongue PPG clip for subjects 1 thru 4 and a neck PPG clip for subjects 5 and 6. BP and BF are blood pressure and blood flow, respectively.

As shown in Figure 1, I‐PTT was determined as the time delay between the proximal BF and distal BP waveforms; PPG‐PTT was determined as the time delay between the proximal and distal PPG waveforms; and PAT was determined as the time delay between the ECG and distal PPG waveforms. The minimum, maximum double derivative, intersection of tangent lines, maximum derivative, and peak of the BF, BP, and PPG waveforms and the R‐wave of the ECG waveform were automatically detected for each beat to establish multiple versions of each time delay (Chiu et al. 1991; Gao et al. 2014). Each version of each time delay was then computed over 1‐min intervals by averaging the middle tertile of the values (for robustness against any artifact and misdetections) and plotted against the corresponding 1‐min averages of diastolic, mean, and systolic BP for each subject. Linear, quadratic, cubic, logarithmic (including an intercept), and reciprocal (including an intercept) functions were fitted to the data points in all of the plots. The degree of fit was quantified using the *R* ^2^ value. The version of each time delay and the function that yielded the best monotonic fit were selected. Finally, the group average *R* ^2^ values of the three time delays were compared for each BP level via one‐way repeated measures ANOVA and the Tukey test for multiple comparisons.

## Results

**Figure 2:**
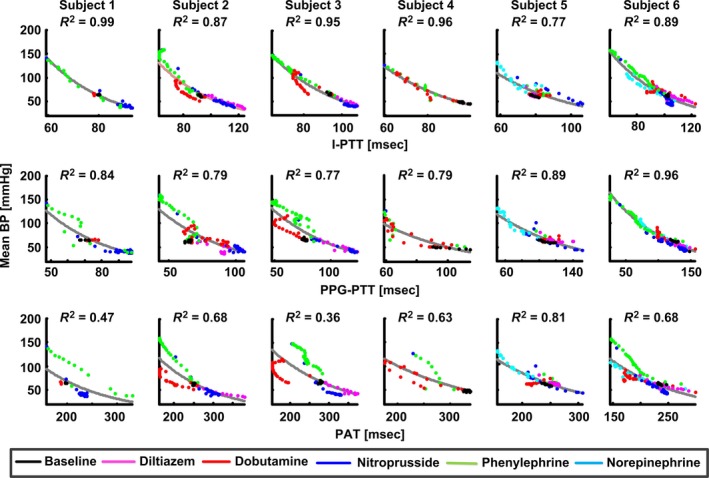
Plots of mean BP versus I‐PTT, PPG‐PTT, and PAT per subject along with best‐fit exponential functions and corresponding *R* ^2^ values.

Figure 2 shows plots of mean BP versus each time delay per subject. The figure includes exponential functions of best fit, and the data points are color‐coded to indicate the hemodynamic drug. The Table shows the individual subject and group average (mean ± SE) *R* ^2^ values between each time delay and each BP level. The *R* ^2^ values generally corresponded to visual assessment of the degree of fitting and therefore served as a good quantitative index of BP tracking ability here.

The BP levels varied widely in each subject. That is, the group averages of the standard deviations of diastolic, mean, and systolic BP were 25 ± 2, 29 ± 2, and 36 ± 2 mmHg, respectively.

The time delays were determined via waveform feet definitions. In particular, I‐PTT was determined as the time delay between the maximal second derivatives of the proximal BF and distal BP waveforms; PPG‐PTT was determined as the time delay between the minima of the proximal and distal PPG waveforms; and PAT was determined as the time delay between the R‐wave of the ECG waveform and the maximal second derivative of the distal PPG waveform. Interchanging the maximal second derivative, minimum, or intersection of tangent lines of the waveforms with each other resulted in only modestly lower *R* ^2^ values for each time delay; however, use of the peak or maximum derivative of the waveforms yielded noticeably smaller *R* ^2^ values for all of the time delays (results not shown).

An exponential function generally provided the best monotonic fit of the data points in the plots of each BP level versus each time delay (results not shown). The exponential function was specifically of the form, BP = A·e^B·PTT^.

As expected, I‐PTT correlated tightly with BP. I‐PTT, in fact, served as a strong marker of diastolic, mean, and systolic BP (group average *R* ^2^ values between 0.86 ± 0.03 and 0.91 ± 0.03) and was never a poor marker of any BP level in any subject (lowest *R* ^2^ value of 0.74).

PPG‐PTT also correlated well with all three BP levels in each subject (group average *R* ^2^ values between 0.81 ± 0.03 and 0.85 ± 0.02). PPG‐PTT was not significantly different from I‐PTT as a marker of each BP level (all P‐values from comparisons of corresponding *R* ^2^ values were above 0.5). While PPG‐PTT appeared to perform best and even better than I‐PTT in the last two subjects with a neck rather than tongue PPG sensor, the improved performance could also be attributable to differences in the employed drugs and their effect in these particular subjects.

Consistent with previous findings, PAT correlated appreciably better with systolic BP (group average *R* ^2^ value of 0.70 ± 0.04) than diastolic and mean BP (group average *R* ^2^ values of 0.49 ± 0.07 and 0.60 ± 0.06, respectively). However, PAT was inferior to both I‐PTT and PPG‐PTT as a marker of all three BP levels (all *P*‐values from comparisons of corresponding *R* ^2^ values were less than 0.001 for I‐PTT and 0.02 for PPG‐PTT). PEP was clearly responsible for PAT's inferior correlation with BP. For example, both phenylephrine (green data points in Fig. 2) and dobutamine (red data points in Fig. 2) increased BP to similar levels. But, phenylephrine increased PEP by 10.4 ± 7.3 msec relative to baseline conditions via enhanced afterload, whereas dobutamine decreased PEP by 30.1 ± 6.0 msec relative to baseline conditions via enhanced contractility. As a result, PAT, which equals the sum of PTT and PEP, was particularly poor as a marker of high BP and could not always adequately track even systolic BP (lowest *R* ^2^ value of 0.51).

## Discussion

In this study, PAT, PPG‐PTT, and I‐PTT were compared as markers of BP during infusions of various hemodynamic drugs in animals. By leveraging an animal model, this study may be the first to compare the ability of noninvasive PTT estimates in tracking BP changes over a wide physiological BP range using I‐PTT measurements as a reference.

More specifically, I‐PTT was determined as the foot‐to‐foot time delay between invasive proximal BF and distal (femoral artery) BP waveforms; PPG‐PTT was determined as the foot‐to‐foot time delay between proximal and distal (slightly distal to the opposite femoral artery) PPG waveforms; and PAT was determined as the time delay between the R‐wave of the ECG waveform and the foot of the same distal PPG waveform (see Fig. 1). Use of the peak or maximal derivative of the waveforms instead of the feet for establishing the time delays compromised their ability to track all BP levels including systolic BP, which occurs near the waveform peaks (results not shown).

An exponential function better fit the data points in the plots of the BP levels versus the time delays than previously proposed functions such as BP = A·ln(PTT) + B or BP = (A/PTT) + B (Mukkamala et al. 2015) (see Fig. 2). This finding could be due to confounding factors that also influence PTT. For example, according to the Bramwell‐Hill equation, PTT not only varies positively with arterial compliance but also inversely with the arterial cross‐sectional area. The arterial cross‐sectional area changes more per a unit change in BP in the low BP regime wherein parallel collagen fibers do not exert tension (Mukkamala et al. 2015). Hence, as BP decreases in the low BP regime, the arterial cross‐sectional area may have been appreciably decreasing so as to cause PTT to increase more per unit change in BP in this regime as compared to the high BP regime.

Consistent with previous studies (Geddes et al. 1981; Pruett et al. 1988; Ochiai et al. 1999; Gao et al. 2014), I‐PTT correlated tightly with BP (see Fig. 2). The group average *R* ^2^ values resulting from the exponential fitting between I‐PTT and diastolic, mean, and systolic BP were between 0.86 ± 0.03 and 0.91 ± 0.03 (see Table 1). In theory, I‐PTT should correlate best with diastolic BP, as it was determined from the waveform feet, which denotes diastolic BP. Since I‐PTT showed similar correlations with mean and systolic BP, all three BP levels were clearly correlated with each other in this study.

**Table 1:** Individual subject and group average correlation values between blood pressure (BP) levels and time delays

*R* ^2^	Subject						
	1	2	3	4	5	6	Mean ± SE
I‐PTT	0.98	0.79	0.88	0.89	0.77	0.87	0.86 ± 0.03
Diastolic BP							
PPG‐PTT	0.79	0.79	0.67	0.78	0.89	0.94	0.81 ± 0.03
PAT	0.31	0.57	0.23	0.48	0.77	0.59	0.49 ± 0.07
I‐PTT	0.99	0.87	0.95	0.96	0.77	0.89	0.91 ± 0.03
Mean BP							
PPG‐PTT	0.84	0.79	0.77	0.79	0.89	0.96	0.84 ± 0.03
PAT	0.47	0.68	0.36	0.63	0.81	0.68	0.60 ± 0.06
I‐PTT	0.93	0.95	0.94	0.94	0.74	0.88	0.90 ± 0.03
Systolic BP							
PPG‐PTT	0.87	0.82	0.87	0.80	0.83	0.92	0.85 ± 0.02
PAT	0.63	0.81	0.51	0.70	0.82	0.73	0.70 ± 0.04

*P*‐value	Diastolic BP	Mean BP	Systolic BP
I‐PTT versus PPG‐PTT	0.76	0.57	0.65
I‐PTT versus PAT	<0.001	<0.001	<0.01
PPG‐PTT versus PAT	<0.005	<0.01	<0.02

The *R* ^2^ values indicate the degree of exponential fitting between each BP level and time delay. The *P*‐values were obtained from the Tukey test for multiple comparisons, as one‐way repeated measures ANOVA comparisons of the three time delays for each BP level always yielded *P* < 0.05. I‐PTT is invasive pulse transit time, PPG‐PTT is photoplethysmography‐pulse transit time, and PAT is pulse arrival time (see Fig. 1 for further details).

Also, consistent with previous studies (Mukkamala et al. 2015), PAT correlated better with systolic BP than diastolic and mean BP (see Table 1). The reason for the result could be that PAT, which includes PEP in addition to PTT, and systolic BP are both determined by the ventricles and arteries. However, PAT was significantly inferior to I‐PTT as a marker of the BP levels including systolic BP (group average *R* ^2^ values of 0.70 ± 0.04 vs. 0.90 ± 0.03; *P* < 0.01). The reason for this result was, not surprisingly, the inclusion of PEP in PAT (see Fig. 2). Interestingly, invasive PAT (the time delay between the R‐wave of the ECG waveform and the foot of the distal BP waveform) was not as good a marker of the BP levels (group average *R* ^2^ values ranging from 0.22 ± 0.09 to 0.37 ± 0.08) as noninvasive PAT (results not shown). This result suggests that the time delay between the distal BP and PPG waveforms was nontrivial and correlated with BP (see below).

Similar to I‐PTT, PPG‐PTT correlated equally well with all BP levels. The group average *R* ^2^ values ranged from 0.81 ± 0.03 and 0.85 ± 0.02 (see Table 1 and Fig. 2). These values were significantly higher than those for PAT even for systolic BP (group average *R* ^2^ values of 0.85 ± 0.02 vs. 0.70 ± 0.04; *P* < 0.02) and not significantly different from those for I‐PTT.

**Figure 3:**
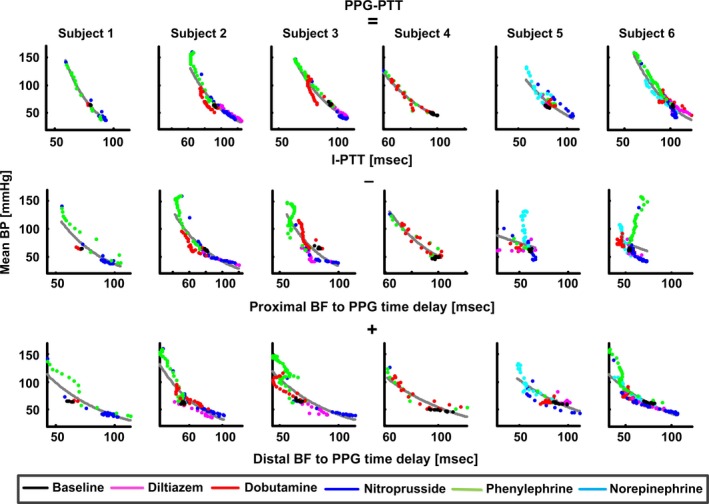
Breakdown of plots of mean BP versus PPG‐PTT into plots of mean BP versus the constituent time delays of PPG‐PTT per subject. The time delays between proximal BF and PPG waveforms and between distal BP and PPG waveforms were significant and dependent on BP. However, the plots of mean BP versus these time delays were similar, so the time delays partially canceled each other out in the formation of PPG‐PTT.

However, PPG‐PTT and I‐PTT were not identical. Figure 3 shows a breakdown of plots of mean BP versus PPG‐PTT into plots of mean BP versus the constituent time delays of PPG‐PTT per subject. As indicated in Figures 1 and 3, PPG‐PTT equals I‐PTT minus the time delay between proximal BF and PPG waveforms plus the time delay between distal BP and PPG waveforms. The plots of mean BP versus the latter two time delays appeared similar, so these time delays may have partially canceled each other out in the formation of PPG‐PTT. However, perhaps counterintuitively, neither was relatively small nor independent of BP. These nontrivial time delays may be caused by viscous effects. In particular, the path for wave travel here (e.g., from the femoral artery to skin vessels slightly distal to the femoral artery) consisted of very small arteries, which are governed by viscous BF. According to Womersley theory, the transit time in such arteries can increase with decreasing vessel radius (Milnor 1982). (It should be noted, however, that this theory has been recently challenged (Painter 2008) Alternatively or in addition, the peripheral arterial wall exhibits a significant viscous response in addition to an elastic response when loaded (Learoyd and Taylor 1966; Mukkamala et al. 2015). Such viscoelastic wall properties cause BP and blood volume to be dynamically related such that PPG, which is a metric of blood volume, can be delayed in time with respect to BP (Bergel 1961; Learoyd and Taylor 1966; Mukkamala et al. 2015). It is also worth mentioning that wave reflection could possibly have contributed to the genesis of the two nontrivial time delays. Finally, the dependency of these time delays on BP could potentially be attributable, at least in part, to the high degree of nonlinearity of the peripheral arterial compliance (see Fig. 3 in [Cox 1978]).

PPG‐PTT was able to track diastolic, mean, and systolic BP in this study because all three BP levels were well correlated here. While this correlation may be typical, there are conditions wherein diastolic and systolic BP do not change in parallel such as central hypovolemia induced by lower body negative pressure (Convertino et al. 2006). In such conditions, PPG‐PTT may only be able to track diastolic BP, as it is determined from the waveform feet. However, based on the results of this study, PAT could possibly be a better marker of systolic BP in these conditions but not as good as PPG‐PTT would be as a marker of diastolic BP.

PPG‐PTT as well as PAT and I‐PTT indicated wave travel time through central arteries here. In most previous studies, wave travel time through peripheral arteries, especially the time delay between ECG and finger PPG waveforms (i.e., finger PAT), was determined (Mukkamala et al. 2015). Since central arteries have less smooth muscle than peripheral arteries, wave travel time through central arteries may be mainly dependent on BP, whereas wave travel time through peripheral arteries may depend on BP and confounding smooth muscle contraction (Mukkamala et al. 2015). For this reason, wave travel time through central arteries may be preferred.

In conclusion, the results of this study suggest that PPG‐PTT should be explored in humans to achieve cuff‐less BP monitoring. Use of ear and toe PPG sensors may be a sensible choice for determining PPG‐PTT. One reason is that PPG waveforms can often be well measured from the ear and toe (2011). Another reason is that wave travel time through central arteries would be determined. Indeed, one previous study showed that PPG‐PTT determined via ear and toe PPG sensors was able to track diastolic BP in surgical patients (Chen et al. 2009). Further human studies are needed to confirm and extend these results. If PPG‐PTT via ear and toe sensors proves sufficient in correlating with BP changes in humans, challenges will remain in calibrating the time delay in units of msec to BP in units of mmHg and creating a practical device. Note that such a device could not be used for chronic BP monitoring without periodic recalibration, as PTT through central arteries changes with aging and disease in addition to BP. Also, note that even if calibration efforts were unsuccessful, PPG‐PTT, by itself, may still permit tracking of relative changes in BP over time periods shorter than aging and disease processes (e.g., within a year) and may thus be useful for guiding antihypertensive therapy in individual patients.

## Conflict of Interest

None.
